# Editorial: Epigenetic regulation in cardiovascular diseases, volume II

**DOI:** 10.3389/fcvm.2023.1166268

**Published:** 2023-03-06

**Authors:** Suowen Xu, Indulekha C. L. Pillai, Christoph D. Rau, Zhihua Wang

**Affiliations:** ^1^ Department of Endocrinology, Institute of Endocrine and Metabolic Diseases, The First Affiliated Hospital of USTC, Division of Life Sciences and Medicine, Clinical Research Hospital of Chinese Academy of Sciences (Hefei), University of Science and Technology of China, Hefei, China; ^2^ Stem Cells and Regenerative Biology Laboratory, School of Biotechnology, Amrita Vishwa Vidyapeetham, Kollam, India; ^3^ Department of Genetics and Computational Medicine Program, The University of North Carolina at Chapel Hill, Chapel Hill, NC, United States; ^4^ Shenzhen Key Laboratory of Cardiovascular Disease, Fuwai Hospital Chinese Academy of Medical Sciences, Shenzhen, China; ^5^ State Key Laboratory of Cardiovascular Disease, Fuwai Hospital, National Center for Cardiovascular Disease, Chinese Academy of Medical Sciences and Peking Union Medical College, Beijing, China

**Keywords:** cardiovasular disease, epigenetics, histone modifications, DNA modification, gene reprogramming

**Editorial on the Research Topic** Epigenetic regulation in cardiovascular diseases, volume II

Cardiovascular disease (CVD) is the leading cause of morbidity and mortality in both developed and developing countries. Precise diagnosis, prevention, and targeted therapies of CVD essentially relies on our deepened understanding of the complex pathophysiology of CVD. The pathogenesis of CVD can be understood at three different levels, i.e., systems level, cellular level and molecular level. The phenotype at the systems level can be explained by changes at the cellular level, while the cellular phenotype is determined by molecular events driven by changes in gene expression patterns. Emerging evidence has unequivocally demonstrated the important role of epigenetics in the regulation of gene expression in various cell types involved in CVD. Epigenetics, by its definition, indicate heritable phenotypes resulting from changes in chromatin without altering DNA sequence. Beyond transcriptional regulation by transcription factors, the expression of genes can be controlled by, among other things, DNA methylation, histone modifications, RNA modifications, and chromatin remodeling. It has been well recognized that epigenetic regulation of gene expression is high dynamic and reversible as various writers, readers, and erasers of epigenetic modification act in concert to regulate gene expression programs related to CVD. Studies elucidating epigenetic regulations in CVD will, in turn, promote novel drug discoveries to treat CVD by targeting epigenetic modifications. In the current research topic, we have collected 5 high-quality studies which include 3 review articles and 2 research articles, which cover research advance and future direction in the epigenetic regulation of CVD, ranging from atherosclerosis to myocardial injury and remodeling.

Atherosclerosis is the pathological basis for panvascular disease including myocardial infarction and ischemic stroke. Atherosclerosis has long been considered as a chronic inflammatory disease, in which polarization of macrophages plays a vital role. Macrophages can be broadly divided into three categories, M0, M1 (pro-inflammatory) and M2 (anti-inflammatory, tissue repairing and pro-fibrotic). Mounting evidence has demonstrated that macrophage polarization plays an important role in the initiation and progression of atherosclerosis. To elucidate the important role of macrophage polarization in atherosclerosis, Yang et al. provided an updated review of DNA methylation and various kinds of histone modifications (e.g., methylation, acetylation, and lactylation) in macrophage polarization, with an aim to developing potential therapies targeting the epigenetic modifiers of M1 to M2 polarization against atherosclerosis.

Endothelial cells are the innermost layer of cells that are directly exposed to circulating blood as well as various types of vascular injury, such as vascular stents. The placement of stents causes damage to the vascular endothelium. In response to vascular injury, circulating endothelial progenitor cells (EPCs) serve as active “craftsman” in the repairing work by a process termed “re-endothelization” to minimize restenosis. However, under diabetic conditions, EPCs number and function is impaired. To decipher the important role of EPCs in vascular injury repair, Arencibia and Salazar performed a datamining analysis of published datasets focusing on the transcriptomic profiles of differentiation of EPCs into mature ECs. After analysis of differentially expressed genes among different dataset, pathway of “AGE-RAGE signaling pathway in diabetic complications” was identified. Further, two up-regulated (IL1B and STAT5A) and two down-regulated (IL6 and MAPK11) genes were identified as hub genes targeted by 5 hub miRNAs. This study implicates these identified hub genes and miRNAs as potential biomarkers of EPCs differentiation and re-endothelization, especially in patients suffering from diabetes.

Cardiac hypertrophy and myocardial remodeling ensuing pressure overload drives heart failure. To obtain a global picture of epigenetics in hypertrophic heart diseases, Lizcano and Bustamante as well as Zhao et al. provided two complementary and comprehensive review of DNA methylation, histone modifications (such as acetylation, methylation, phosphorylation and ADP-ribosylation etc.), non-coding RNAs (such as miRNA and long non-coding RNAs), chromatin remodeling factors (such as SWI/SNF complex) and RNA modifications in regulating multiple cellular events in cardiac hypertrophy. Epigenetic drugs targeting these epigenetic modifications in CVD are also reviewed to make drugging epigenetic modifications possible. In-depth comprehension into epigenetic modifications in human cardiac hypertrophy will pave the way for the development of novel therapeutic approaches. Myocardial ischemia/reperfusion (I/R) injury leads to myocardial infarction. To explore key potential therapeutic targets for treating myocardial I/R injury, Meng et al. performed a datamining study of published datasets and identified CCL21 and its receptor GPR174/CCR7 were increased following myocardial I/R injury in mice. Furthermore, the activated CCL21-GPR174 signaling was located on the cardiac fibroblasts of the mouse myocardium with I/R injury. This study revealed CCL21-GPR174/CCR7 signaling in cardiac fibroblasts as a potential therapeutic target underlying myocardial I/R injury. Further studies are warranted to unravel whether blocking CCL21-GPR174/CCR7 signaling pathway in cardiac fibroblasts will confer cardioprotection in mice by conditional knockout mice, and eventually in human patients.

Taken together, the present research topic (***Epigenetic Regulation in Cardiovascular Diseases, Volume II***), along with its preceding volume (superlink), demonstrates the important role of epigenetics in CVD, including ways in which epigenetics could lead to novel therapeutic options. Since some epigenetic drugs targeting DNA methylation and histone modifications are currently in clinical use or in different stages of clinical development in cancer therapeutics, it remains to be seen whether these epigenetic drugs can be repurposed in cardiovascular therapeutics. Last but not least, in light of the emerging role of RNA methylation in CVD (such as atherosclerosis), future studies are warranted to provide proof-of-concept that RNA methylation can also be therapeutically targeted similar as DNA and histone modifications.

